# Naturalistic exploratory study of the associations of substance use on ADHD outcomes and function

**DOI:** 10.1186/s12888-021-03263-6

**Published:** 2021-05-12

**Authors:** Benjamin MacDonald, Joseph Sadek

**Affiliations:** 1 Department of Medicine, Dalhousie University, Halifax, NS Canada; 2 Department of Psychiatry, Dalhousie University, Halifax, NS Canada

**Keywords:** Attention-deficit/hyperactivity disorder, Substance abuse, Cognition, Psychosocial functioning

## Abstract

**Background:**

Although Attention Deficit Hyperactivity Disorder (ADHD) is associated with an increased risk of substance use disorder (SUD), existing literature on how SUD interacts with ADHD outcomes is limited. This study investigates whether SUD among individuals with ADHD is associated with worse ADHD outcomes and prognosis, and the association between overall functioning and SUD. In addition, we seek to understand whether heavy cannabis use is a better predictor of poorer outcomes compared to SUD status alone.

**Method:**

We conducted a retrospective analysis on 50 ADHD patient charts, which were allocated based on SUD status. Subgroup analysis was performed on the total sample population, with allocation based on heavy cannabis use. Mann-Whitney and Chi-Square tests were used for both the primary and subgroup analyses.

**Results:**

SUD status highly correlated with more ADHD-related cognitive impairments and poorer functional outcomes at the time of diagnosis. ADHD patients with comorbid ADHD-SUD scored significantly lower (*p* = < 0.0001) on objective cognitive testing (Integrated Auditory and Visual Continuous Performance Test (IVA/CPT)) than ADHD patients without SUD. The correlation with poorer ADHD outcomes was more pronounced when groups were allocated based on heavy cannabis use status; in addition to significantly lower IVA/CPT scores (*p* = 0.0011), heavy cannabis use was associated with more severe fine motor hyperactivity and self-reported hyperactivity/impulsivity scores (*p* = 0.0088 and 0.0172, respectively).

**Conclusion:**

Future research is needed to determine how substance abuse can be a barrier to improved ADHD outcomes, and the effect cannabis and other substances have on cognitive function and pharmacotherapy of ADHD.

## Background

Attention Deficit Hyperactivity Disorder (ADHD) is a neurodevelopmental disorder characterized as a persistent pattern of inattention, hyperactivity, and/or impulsivity [1]. The understanding of the etiology of ADHD continues to evolve in response to research on the neurobiology, genetics, and clinical nature of ADHD. Decades of neuroimaging research have shown multiple ADHD-related abnormalities in brain structure and function, and suggests that ADHD may in part be due to delayed or disrupted neurodevelopment and maturation. These brain regions have been shown to be responsible for higher order executive functions; including executive control over behavior, attention, social cognition, and networks supporting primary sensory and motor functions [2, 3]. Childhood ADHD is associated with several functional impairments including reduced school performance and academic attainment, and social rejection [1]. In adults, ADHD is associated with poorer occupational performance and attainment, attendance, interpersonal conflicts, as well as a higher probability of unemployment and substance abuse [1]. Substance use disorder (SUD) is characterized by a problematic pattern of substance abuse regardless of short and long term consequences, leading to clinically significant impairment or distress [1]. While ADHD and SUD are different disorders, they often coexist with each other. Children with ADHD are estimated to be 2.64 times more likely to develop SUD, and individuals with ADHD are at an increased risk of substance misuse/dependence, including cannabis misuse [4–6]. A study that followed 579 children with ADHD and 258 age- and sex-matched children without ADHD, found that the ADHD group had earlier initiation- and faster escalation- of substance use in adolescence. Interestingly, the ADHD group had more weekly cannabis use (32.8% vs 21.3%), and double the rate of daily cannabis use (22.4% vs 10.9%) [7]. Cannabis use in general seems to be increasing among adults and adolescents [8].

The acute effects of cannabis on cognition has been investigated, and has been shown to impair the following; verbal memory and learning, working memory, attention (task and dose dependent), inhibition, and psychomotor function [9]. A study investigating whether these neurocognitive deficits persist, showed that heavy cannabis users (daily use) - relative to light users - had persisting deficits in verbal and visual memory, executive functioning, visuo-perception, psychomotor speed, and manual dexterity, even after 28 days of abstinence [10]. Cannabis use in youth, regardless of ADHD diagnosis, has shown that it may lead to poorer performance on tasks that require attention functioning, decreased verbal working memory, and decreased executive functioning [8, 11, 12]. A study investigating the effects of cannabis on ADHD patients’ response inhibition, showed that cannabis use was significantly associated with a slower continuous performance test (CPT) hit rate response [13]. A large (1037 participants) prospective study, which followed participants from birth to age 38, found that cannabis use was associated with more global neuropsychological impairments, including IQ, and found specific deficits in executive functioning, sustained attention, verbal list learning, and psychomotor speed. These effects were more prominent in adolescent-onset users, where more persistent use is associated with greater decline. This study is unique, as its prospective design was able to control for premorbid neuropsychological deficit and years of education, and showed that among adolescent-onset (and former persistent users), impairment was still evident even after cessation of use for 1 year or more [14]. Additionally, cannabis misuse results in negative changes in brain regions associated with response inhibition [2, 15], and these declines in neuropsychological functioning are thought to be more likely to manifest among daily (or almost daily) cannabis users [16]. Another study showed evidence which suggests that early onset of regular cannabis use may disrupt neuromaturation, especially in networks responsible for executive functions and rewards [17]. Interestingly, a meta-analysis investigating whether these brain function altercations persist on fMRI with abstinence, showed that these altercations clearly persist past 25 days abstinence in adolescent users – despite no detectable THC in urine [18]. Since the endocannabinoid system plays a role in neuroplasticity [19, 20], and neuromaturation is prevalent during adolescence [21], it is plausible that exogenous cannabinoids alters the neurodevelopmental maturation during this period – suggesting a period of vulnerability [8].

These findings are fairly consistent across reviews and meta analyses, all of which identify specific subgroups of cannabis user that are at risk of more severe neurocognitive deficits, such as early (adolescent) use onset [8, 9, 17, 21–23], and extent of exposure – including chronic use [18, 23–25], and regular (daily or almost daily) use [8, 16, 22, 24]. The concept that the neurocognitive deficits differs in severity based on specific cannabis use characteristics (age of onset, duration, frequency and quantity), is important to understand when interpreting existing literature on these topics, as not all studies control for these factors. As an example, a co-twin design study [26] (n of 856), which investigated the causal effects of cannabis on cognition, found between-family significance for measures of intelligence and executive functioning, but not after accounting for other substance use. However, only 16 participants (less then 2% of the total population sample) reported daily use, and therefor might not be generalizable to that population. It is important to note that only one effect – between age 17 cannabis frequency and executive function at age 23 - remained significant across all within-family levels and after accounting for other substance use.

Despite the overwhelming amount of studies that support the harms of cannabis use on ADHD outcomes, there are still studies that investigate the therapeutic use of cannabinoids in ADHD patients [27] and show that cannabis is perceived to be beneficial for some individuals with ADHD [28]. It is possible that the ratio of THC relative to CBD may account for some of the discrepancy of the findings among some of the studies [29]. In addition to discordance around hypotheses and research questions in current literature on cannabis use and ADHD symptoms, there are several limitations to these studies that precludes the ability to establish causation. These limitations include cross-sectional studies with modest sample sizes, and the heterogeneity in the dichotomous designation of cannabis use (i.e. Cannabis use thresholds used to allocate participants to study groups). Although cross sectional studies played a significant role in the advancement of research in this area, they do not control for confounding variables. This includes the inability to assess how changes in substance use prospectively influence ADHD-related cognitive functioning, and the inability to control for differences in baseline characteristics. Future studies should use cotwin-controlled designs or prospective designs which control for daily users and/or heavy use in adolescence, to better control for potential confounds - as these studies are rare and have low sample sizes [6, 14, 18, 30, 31]. Another challenge in this area of research is the outcome measures used, where studies that use self-report outcome measures are at risk of response bias. There is only a small amount of literature that specifically investigates SUD (and cannabis use alone) on objective cognitive testing and all-around functioning in society.

The Integrated Auditory and Visual Continuous Performance test (IVA/CPT) was the objective measure used in our study, which is a standardized test that has been validated for diagnostic accuracy in comparison to clinician diagnosed ADHD, with EEG correspondence. The IVA/CPT computes a sustained auditory and visual attention quotient (SAAQ and SVAQ, respectively), response quotient, and fine motor hyperactivity quotient. The attention quotient consists of a summary of separate audio and visual measures of vigilance (a measure of inattention based on omission of errors), focus (total variability of mental processing speed for correct responses), and speed (average reaction time for correct responses). The response control quotient is comprised of separate auditory and visual scores for prudence (a measure of impulsivity and response inhibition based on commission errors), consistency (a measure of one’s ability to stay on task by variability in response times), and stamina (a measure of sustained effort over time by comparing mean reaction times of the first 200 correct responses vs last 200 correct responses) [32].

This study is unique as it is one of the few studies that specifically focuses on both objective cognitive measures and psychosocial functioning. Our explorative study seeks to understand: 1) How do the cognitive profiles of patients who have ADHD and SUD differ from patients with ADHD without SUD, 2) Does SUD among adults in a naturalistic outpatient community setting result in more functional impairment and worse ADHD outcomes, and 3) how do the cognitive profiles of ADHD patients differ based varying cannabis use.

## Methodologies

### Research design

The study is a retrospective analysis and chart review of the first 50 patients with a new ADHD diagnosis, between the dates of January 1st, 2017 and June 1st, 2019 – the first 25 ADHD patients without SUD, and the first 25 ADHD patients with SUD.

Every patient had been assessed and diagnosed by a psychiatrist at an outpatient psychiatric clinic that specializes in ADHD, after a full psychiatric evaluation. In addition, all participants completed a set of questionnaires to gain more information on their personal and psychiatric history, and level of functioning. All patients were tested by the IVA/CPT.

The independent variables in the study are ADHD patients with substance use disorder (SUD group), and ADHD patients without substance use disorder (Non SUD group).

The dependent variables are as follows; demographics (age, gender), suicide risk level, medical comorbidities, alcohol and substance use history, psychiatric comorbid disorders, IVA/CPT score, number of non-valid IVA/CPT scores, parental breakups, family history of substance use disorder, childhood adversity events such as sexual and physical abuse, highest attained education level, interactions with the law, and employment status. The dependent variable data was collected from diagnosed patients through the many questionnaires’ given to all patients at the clinic. These dependent variables function as markers for ADHD response and function. They served as a way to gain more background information on each patient, and to better identify potential confounding variables, which facilitated proper analysis and interpretation of the results. Subgroup analysis was performed within the total sample population, and compared heavy cannabis users (CU group) with non-heavy cannabis users (Non CU group).

### Participants

Participants were allocated to one of two groups; 1) participants with ADHD who have DSM-5 defined SUD, and 2) participants with ADHD who do not have DSM-5 defined SUD.

Inclusion criteria: newly diagnosed patients with ADHD who are under the care of a psychiatrist specializing in ADHD, between the dates of January 1st, 2017 and June 1st, 2019.

Exclusion criteria: Patients who got diagnosed with ADHD; 1) without an Integrated Auditory and Visual Continuous Performance test score, 2) Whose reported substance use is too ambiguous to determine substance use status (in accordance with the DSM-5 Substance use disorder and intoxication criteria), or have skipped section(s) in the substance use questionnaire. The number of excluded charts are small, and would not affect statistical analysis and results. Additionally, CPT results were not known for participants who were excluded due to incomplete substance use questionnaire. Therefore, CPT results did not influence the decision to exclude participants due to incomplete questionnaires.

### Participant recruitment and allocation

In order to obtain our target of 50 patient charts in total, the supervising investigator reviewed patient charts sequentially (by decreasing date), starting on June 1st, 2019 until 25 charts in each group are identified. The maximum date range for participant recruitment was between the dates of January 1st, 2017 to June 1st, 2019. All substances in the DSM-5 SUD criteria were used to allocate patient charts based on substance use status except for nicotine and caffeine, as these are substances that can enhance ADHD-related cognition [1], and would serve as a confounding variable in our analysis. If a patient did not meet DSM-5 SUD criteria for all other measured substances, but used nicotine or caffeine, they were allocated to the Non SUD group. Alternately, if a patient met DSM-5 SUD criteria for substances other than nicotine and caffeine, and used nicotine and/or caffeine, they were allocated to the SUD group. Data on nicotine use was collected and compared between groups in order to control for this variable. Data on caffeine use was not collected as the substance use questionnaire wasn’t designed to do so.

When patient charts satisfied inclusion and exclusion criteria, and met DSM-5 criteria for SUD, they were allocated to the SUD group. When patient charts satisfied inclusion and exclusion criteria, and did not meet DMS-5 criteria for SUD, they were allocated to the Non SUD group. The maximum number of charts that could be reviewed, in order to attain our goal of 25 charts per group, was set at 500 charts. This selection process was chosen as a way of randomization, and to control for selection & allocation bias, where participant selection was dependent on when they got diagnosed, not on the choice of the researchers.

After the primary analysis, Subgroup analyses was performed. The subgroup analysis compared heavy cannabis users (CU group) with those who either did not use cannabis, or light users of cannabis below the heavy cannabis use threshold (Non CU group). We chose a more conservative threshold as to whether one’s cannabis use met heavy cannabis use status, as the substance was recently legalized in the country. Heavy cannabis use in this study was defined as use 3 times a week or greater, or last use within 72 h at the time of diagnosis among weekly users. We chose this method to capture the cannabis users that are at higher risk (daily or near daily use) of neurocognitive deficits, based on numerous meta-analyses [8, 16, 22, 24]. We set our threshold as use 3 times a week or greater, as this population would be expected to still have relatively high cannabinoid levels in their system [33], allowing us to capture any cognitive deficits due to the acute effects of cannabis. Additionally, there was only 4 participants in the study who used cannabis 2–6 times per week, and there was no use of cannabis within 72 h in patients that used cannabis less than weekly.

### Outcome measures

#### Measures of cognition and ADHD outcomes

Measure of ADHD outcomes were classified and calculated based on objective cognitive testing or subjective patient report. Objective cognitive testing measures were calculated based on participant’s completion of the Integrated Visual and Auditory Continuous Performance task. The following objective measures are computed by the IVA/CPT; Sustained Auditory & Visual Attention Quotients (SAAQ and SVAQ, respectively), and Fine motor Hyperactivity. Fine Motor Hyperactivity measures off-task impulsive fine motor activity with the mouse, and is computed as a severity score (none, mild, moderate, severe and extreme). Severity score on ADHD symptoms were calculated based on self-reported symptoms consistent with the DSM-5 diagnostic criteria for Inattention and Hyperactivity/Impulsivity. Both hyperactivity/impulsivity and inattention measures were ranked out of 9 – if participants meet any of the 9 symptoms in each checklist, then they were given a point for the respective symptom. For both Inattention and Hyperactivity/Impulsivity checklists, if adult participants had 5 points out of 9 or less, then their symptom severity is mild (given a score of 1). If 6 or 7 points out of 9 then symptom severity is moderate (given a score of 2), and if 8 or 9 points out 9, then symptom severity is severe (given a score of 3).

#### Measures of DSM-5 diagnoses

All patients were assessed by a psychiatrist, whom used DSM-5 Criteria to diagnose the following; ADHD, SUD, major depressive disorder, generalized anxiety disorder, borderline personality disorder. Participants were given questionnaires for all of the above disorder, which were designed based off of DSM-5 symptom criteria. The completed questionnaires were then used in conjunction with the psychiatrists history and mental status exam, in order to confirm these diagnoses, and to ensure all DSM-5 criteria were met.

#### Education level

Highest attained education level was ranked as follows: 1 = less then grade 10, 2 = grades 10–12, 3 = completion of grade 12, 4 = Community college, 5 = Bachelor’s degree, 6 = completion of Master’s degree, 7 = Doctorate degree.

#### Suicide risk

Was graded as either mild, moderate or severe, and was given the numerical values 1, 2 and 3 respectively. Participants were ranked based on the rules of The Nova Scotia Tool for Suicide Risk assessment. Patients were ranked as severe if they had an active plan or intent of suicide with ongoing suicidal ideation. Participants were moderate if they had suicidal ideation, multiple risk factors but not current intent nor plan. Participants were low risk if they had no history of cutting or suicide attempts.

#### Personality disorder and personality traits

Personality disorder and traits were computed based on responses to the personality disorder questionnaire. Each question represents a DSM-5 symptom criteria for a given personality disorder where participants circle either ‘yes’, ‘maybe’, or ‘no’. Participants would meet DSM-5 symptom criteria if they circled ‘yes’, and would not meet the DSM-5 symptom criteria if they circled ‘maybe’ or ‘no’. If the questionnaire contained all DSM-5 symptom criteria for a given personality disorder, and a participant checked off all the symptoms required for a diagnosis of a given personality disorder, then they were said to meet DSM-5 diagnostic criteria for that personality disorder. If the questionnaire contained most DSM-5 symptom criteria for a given personality disorder – but not enough to be diagnostic - then they were said to have personality traits for that personality disorder. Outcome measures for personality traits were analyzed as a ratio of total symptoms checked off for a given personality disorder, divided by total symptoms for that personality disorder.

#### Substance and alcohol use

The substance use questionnaire asks about the daily amount, frequency, and date of last use for the following substances; alcohol, cannabis, hallucinogens, stimulants, cocaine, pain medication not prescribed, and barbiturates. Nicotine use was inquired by checking a yes or no box depending on whether one currently uses it, with a space for the participant to elaborate if they feel necessary. Caffeine use wasn’t collected. These questionnaires were used in conjunction with the patient history to establish a DSM-5 diagnosis of SUD.

#### Statistical analysis

T tests and Chi squared tests were used to determine statistical significance between SUD status and measures of ADHD outcome and function. Odds ratios were calculated for each comparison. The same tests were used for the subgroup analysis.

## Results

### Demographics

Table 1 provides demographic information and statistical tests (Mann-Whitney and Chi-Square test) for both primary analysis groups (SUD vs Non SUD) and secondary analysis groups (CU group vs Non CU group). This table also provides the subject count in each group. The groups were not statistically different in terms of age, gender and confounding medication use.

**Table 1:** Demographics

Outcome measures	SUD *n* = 25	Non SUD *n* = 25	CU *n* = 22	Non CU *n* = 28	*P* values	
					SUD vs Non SUD	CU vs Non CU
Median age (25th /75th percentile)	29 (23.5/ 36.5)	30 (28/37)	29.5 (22.7 /36.2)	29.5 (24.5 /39.5)	0.821	0.519
Gender	M:16	M:11	M: 14	M:13	0.156	0.226
	F:9	F:14	F: 8	F: 15		
Confounding meds						
Nicotine	Yes: 9	Yes: 4	Yes: 8	Yes: 5	0.107	0.139
Benzodiazepines PRN	Yes: 2	Yes: 4	Yes: 1	Yes: 5	0.663	0.318

### Substance use

Table 2 provides information around substance use frequency, which shows how many participants used a given substance according to frequency.

**Table 2:** Substance use

Substance	Frequency	SUD	Non SUD
Cannabis	Daily	15	0
	2–6 times per week	4	0
	Weekly-Monthly	3	5
	2–6 times per year	2	5
	Yearly or never used	1	15
Alcohol	Daily	3	0
	2–6 times per week	1	1
	Weekly	4	2
	1–3 times per month	11	5
	2–6 times per year	2	8
	Yearly or never used	2	9
	Frequency not reported	2	n/a
Cocaine	Use within 1 year	13	0
	History of use (> 1 year)	7	2
	Never used	5	23
Hallucinogens	Use within 1 year	5	0
	History of use (> 1 year)	7	2
	Never used	13	23

Cannabis was the most commonly used drug for the SUD group, whereas alcohol was the most common substance used in the Non SUD group – although alcohol use was still higher for the SUD group compared to the Non SUD group. Cocaine and Hallucinogen use was the least used substance in both groups in terms of frequency.

### IVA/CPT primary analysis

In order to test our hypothesis that Substance use among the ADHD population results in worse ADHD outcomes, statistical analysis (Mann-Whitney Non Parametric T test) was done between SUD groups and Non SUD groups. Table 3 provides the statistical results around objective and subjective cognitive testing between SUD and Non SUD groups, subject counts, and median values. The results indicate that the SUD group had poorer performance and differed significantly for all IVA/CPT measures. The groups did not differ statistically for the reported number of ADHD symptoms. All measures had a sample size of 25 for each group, except for IVA/CPT SAAQ and SVAQ where quotients were excluded if the computers detected idiopathic or random responding errors, and calculated a non-valid IVA/CPT score. There was one non-valid IVA/CPT score in each group.

**Table 3:** Cognitive testing

Outcome measures		*P* value	Medians (n)	
			SUD	Non SUD
Objective measures via IVA/CPT	IVA/CPT SAAQ	0.006**	44.5 (24)	80 (25)
	IVA/CPT SVAQ	0.0001***	18 (25)	81.5 (24)
	IVA/CPT Average	< 0.0001***	38 (25)	83 (25)
	Fine Motor Hyperactivity	0.095	2 (25)	1 (25)
Subjective DSM-5 ADHD questionnaire scores	Inattention Symptoms	0.056	2 (25)	1 (25)
	Hyperactivity & Impulsivity Symptoms	0.284	1 (25)	1 (25)

*P ≤ 0.05,** *P* ≤ 0.01), ***P ≤ 0.001

### Comorbid disorders comparisons

Table 4a provides the statistical results for various psychiatric related measures between SUD and Non SUD groups. Mann-Whitney Non Parametric T test was used to compare median scores between groups. This table shows that the SUD group had significantly more traits consistent with borderline and antisocial personality disorder. These differences were not found for Cluster C personality disorder traits. Table 4a also shows that the SUD group population had a significantly higher suicide risk. One participant in the Non SUD group was excluded in the analysis of personality traits, as they were in late adolescence and therefore did not receive the DSM-5 personality trait questionnaire.

**Table 4:** Psychiatric data

A			
Outcome measures	*P* value	Medians (n)	
		SUD	Non SUD
Borderline personality traits according to DSM-5 criteria	0.015*	0.385 (25)	0.154 (24)
Antisocial personality traits according to DSM-5 criteria	0.012*	0.25 (25)	0 (24)
Cluster C personality traits according to DSM-5 criteria	0.188	0.4 (25)	0.2 (24)
Suicide severity risk	0.034*	1 (25)	1 (25)
Major depressive disorder severity according to DSM-5	0.001***	3 (25)	2 (25)
B			
Outcome measures	*P* value	%Yes (n)	
		SUD	Non SUD
Borderline personality disorder according to DSM-5 criteria	0.0147*	44 (25)	12.5 (24)
Generalized anxiety disorder according to DSM-5 criteria	0.0087**	80 (25)	44 (25)
History of self harm	0.145	48 (25)	28 (25)
Past psychiatric hospitalizations	0.269	24 (25)	12 (25)
Family history of SUD	0.874	44 (25)	32 (25)
Previous psychotherapy	0.564	64 (25)	56 (25)

*P ≤ 0.05,** *P* ≤ 0.01), ***P ≤ 0.001

Table 4b provides the statistical results for dichotomous variables relating to psychiatric health, and therefore Chi-square tests were used to calculate significance. This table shows that more patients in the SUD group met DSM-5 diagnostic criteria for borderline personality disorder and generalized anxiety disorder. There were no statistically significant differences between groups for the following self-reported measures: history of self-harm, in-patient psychiatric hospitalizations, family history of SUD, and previous psychotherapy. As past psychiatric hospitalizations had an expected cell count less then 5 on the Chi-Square, Yates Continuity Corrected Chi-Square was also done to determine a corrected *P* value of 0.462. One participant in the Non SUD group was excluded in the analysis of borderline personality disorder diagnosis, as they were in late adolescence and therefore did not receive the DSM-5 personality disorder questionnaire.

### Level of functioning and adversity factors

In order to test our hypothesis that comorbid ADHD and SUD results in poorer functional impairment within society, data pertaining to violence, interactions with law, academic and occupational achievement and trauma history was analyzed (Table 5). Chi-square tests were used for all variables to calculate statistical significance, except for ‘highest attained education level’ where the Mann-Whitney test was used, as we were comparing medians.

**Table 5:** Societal functioning and adversities

Outcome category	Outcome measures	*P* Value	Percentage Yes (n)	
			SUD	Non SUD
Violence related	Got in Trouble due to Temper/Violence	0.008**	40 (25)	8 (25)
	Drinking/Drugging Leads to Violence	0.004**	28 (25)	0 (25)
Interactions with law	Previous Charges	0.021*	28 (25)	4 (25)
	Previous Arrests	0.123	24 (25)	8 (25)
	Previous Jail time	0.552	8 (25)	4 (25)
Reported abuse	Physical Abuse	0.047*	36 (25)	12 (25)
	Sexual Abuse	0.185	32 (25)	16 (25)
Academic and occupational achievement	Failed Grades: 1 to 12	0.059	40 (25)	16 (25)
	Dropped out of College or University	0.091	20 (25)	4.17 (24)
	Didn’t Complete High school	0.029*	32 (25)	7.69 (25)
	Academic Difficulty	0.087	56 (25)	32 (25)
	Highest attained Education	0.01*	Median: 3	Median: 4
	Current Employment	0.733	80 (25)	76 (25)
Regarding parental relationships	Poor/No Relationship with parents	0.0007***	45.8 (24)	4 (25)
	Parental Split During Childhood/Adolescence	0.564	44 (25)	36 (25)

*P ≤ 0.05,** *P* ≤ 0.01), ***P ≤ 0.001

Significance was found for measures relating to violence, charges, physical abuse, and poor relationship with parents. In terms of academic and occupational achievement, significance was found for not completing high school, and the highest attained education level. The SUD group had a median education level at grade 12, whereas those in the non SUD group had a median college degree education level. History of failing grade, academic difficulty, and dropping out of college/university were approaching significance at an alpha level of 0.05, and met significance with an alpha level of 0.1.

As the event rate for some variables were low, the Yates continuity chi-square was used if any cell in the chi-square had an expected count less than 5. These variables, along with their corrected *P* values, are as follows; Drinking/Drugging Leads to Violence (*P* = 0.014), Previous Charges (*P* = 0.054), Previous Arrests (=0.247), Previous Jail time (*P* > 0.99), Dropped out of College or University (*P* = 0.209), and Current Employment (*P* > 0.99).

### Subgroup analyses

Although subgroup analyses can be difficult to interpret, we elected to compare heavy cannabis users (CU group) with non-heavy cannabis users (Non CU group). Table 6 provides information on the influence of heavy cannabis use on ADHD outcomes and function. Participants in both SUD and Non SUD groups were allocated to the CU subgroup if they used cannabis 3 or more times per week, or used within 72 h of being diagnosed with ADHD among weekly users. If not, the participant would be allocated to the Non CU subgroup.

**Table 6:** Cannabis subgroup analysis

Outcome Measures	*P* value	Medians (n)	
		CU	Non CU
IVA/CPT SAAQ	0.012*	43 (21)	77 (28)
IVA/CPT SVAQ	0.004**	12 (22)	80 (27)
IVA/CPT Average	0.001***	38.75 (22)	80.5 (28)
Fine motor hyperactivity	0.009**	2 (22)	1 (28)
DSM-5 ADHD inattention symptoms	0.071	2 (22)	1 (28)
DSM-5 ADHD hyperactivity & impulsivity symptoms	0.017*	1 (22)	1 (28)
Suicide risk	0.004**	2 (22)	1 (28)
Education level attainment	0.031*	3 (22)	4 (28)
Borderline personality disorder according to DSM-5 criteria	0.003**	% yes: 50	% yes: 11.1
Antisocial personality traits according to DSM-5 criteria	0.026*	0.25 (22)	0 (27)
Cluster C personality traits according to DSM-5 criteria	0.598	0.4 (22)	0.4 (27)
DSM-5 defined major depressive disorder severity	0.001***	3 (22)	2 (28)

*P ≤ 0.05,** *P* ≤ 0.01), ***P ≤ 0.001

The CU group differed significantly from the Non CU group by having more impairment in objective (IVA/CPT) and subjective (self-reported ADHD symptoms) cognitive functioning. Fine motor activity and Hyperactivity/impulsivity symptoms were significantly higher in the CU group – both of which were not seen when comparing groups by SUD status. No significant differences were found when comparing inattention symptoms. Like the comparison of SUD groups, the CU subgroups had significantly poorer performance on IVA/CPT, higher suicide risk, poorer education attainment, more borderline and antisocial personality traits, and higher major depression disorder severity.

## Discussion

**Fig. 1:**
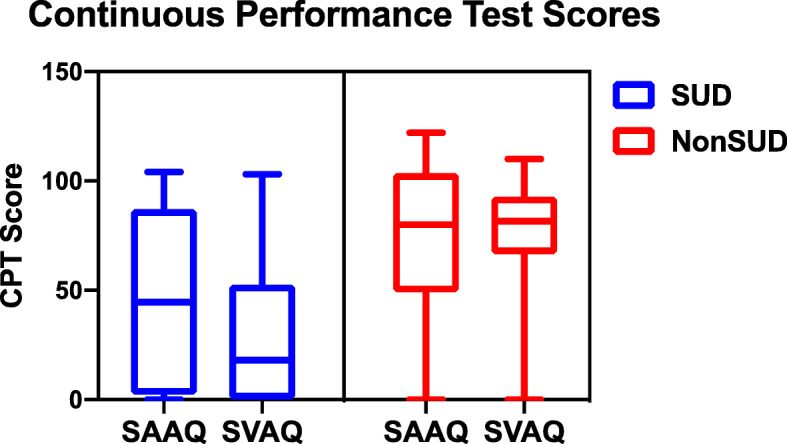
Objectve Sustained Auditory & Visual Quotients  (SAAQ and SVAQ, respectively) calculated from IVA/CPT, and shows a Box and Whisker plot with min/max range, interquartile ranges, and medians

We aimed to investigate how substance abuse among the ADHD population may interact with different outcomes measures, and how the profiles of ADHD patients differ according to SUD status. Our results show that according to IVA/CPT results between groups (Fig. 1, Table 3), SUD status is highly correlated with more impaired ADHD-related cognitive outcomes at the time of diagnosis, to both auditory and visual information.

**Fig. 2:**
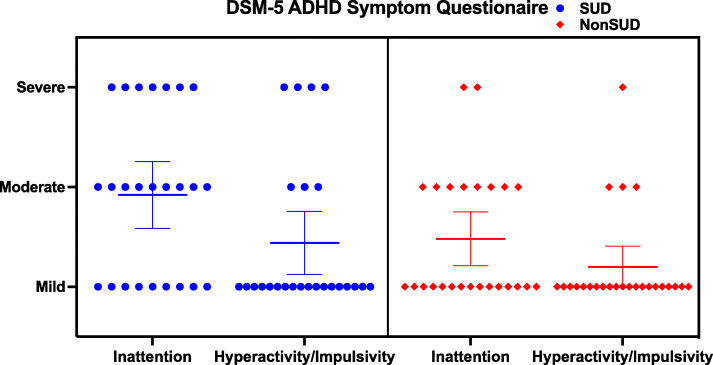
Subjective ADHD Inattention and Hyperactivity/Impulsivity symptom scores, based on participants response to the DSM-5 ADHD Symptom Questionaires. Consists of a Scatter Plot graph, with 95% confidence interval around mean scores

No significant difference was found between groups for reported number of ADHD symptoms (Fig. 2, Table 3). The SUD group had a median inattention severity of moderate while the Non SUD group had a median Inattention severity of mild. As for Hyperactivity/Impulsivity symptoms, both groups had a median severity of mild, although the SUD group had 4 participants with severe symptoms, and the Non SUD group had 1 (Fig. 2).

**Fig. 3:**
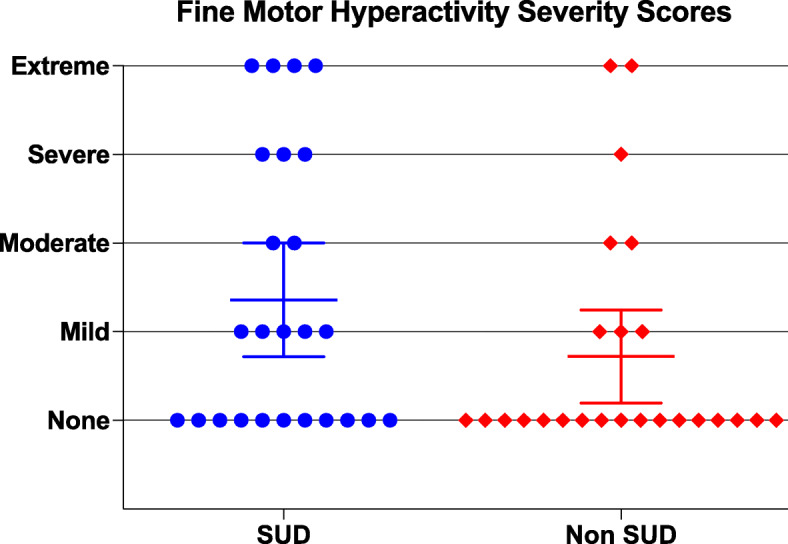
Objective Fine Motor Hyperactivity calculated from IVA/CPT, and shows a scatter plot graph with 95% Confidence Interval around mean scores

Although no significant differences were found for fine motor hyperactivity (Fig. 3, Table 3), significance was trending towards the SUD group having more impulsive fine motor activity. The median severity scores were mild and none for the SUD and Non SUD group respectively, and more participants in the SUD group had extreme and severe symptom severity.

Subgroup analyses investigating heavy cannabis abuse shows even more impairment in objective and subjective ADHD outcomes (Table 6). Significance was found for IVA/CPT SAAQ and SVAQ scores, fine motor hyperactivity, and subjective hyperactivity/impulsivity symptoms. Like SUD status, subjective Inattention symptoms were not significant, but were trending towards significance.

These results suggest that SUD status at diagnosis predicts poorer ADHD outcomes and prognosis. These results bring up the question of how ADHD and SUD interact with one another, and the other factors (genetic, epigenetic, neurodevelopmental, and environmental) that interplay to produce a more severe ADHD phenotype. Are individuals with severe ADHD symptoms (i.e. more deficits in response inhibition, less able to engage in future goal-oriented behaviour, and impulsivity) more likely to abuse substances [34], and/or do the substances themselves directly impair cognition or neurodevelopment through pharmacologic means? Some researchers suggest that youth with ADHD are more likely to initiate substance use earlier, escalate to more frequent substance use, and engage in binge drinking by adult [35, 36]. Another explanation is that the ADHD patients with comorbid disorders such as anxiety or mood disorders, are more likely to abuse substances to the point of meeting SUD criteria [37]. Additionally, mood and anxiety disorders are also highly comorbid with SUD, with co-occurrence lifetime rate of 40.3% for major depression [38], and 29.9% for anxiety disorders [32, 39, 40]. Some of the symptoms of anxiety and mood disorders can overlap with ADHD [32, 41–43].

Studies investigating the effect cannabis has on brain structure and function shows that cannabis use is associated with altered brain structure and function [2, 8, 15, 17, 18, 44, 45, 46]. Our results support that notion, as we found more cognitive impairments and poorer ADHD outcomes when groups were allocated based on heavy cannabis use as appose to substance use status (which included other substances such as alcohol).

Table 4 presents results relating to psychiatric data, by comparing medians between groups with continuous data (Table 4a) and contingency data with dichotomous variables (Table 4b). Results indicate that the SUD group had a higher prevalence of generalized anxiety disorder and borderline personality disorder, a higher severity of major depression, higher suicide risk, and more borderline and antisocial personality traits. Our study shows that the prevalence of comorbid BPD and ADHD in our total sample is 28.6%. The SUD group had significantly more comorbid ADHD and BPD than the Non SUD group (44% versus 12.5% comorbidity). The prevalence of comorbid BPD and ADHD in other studies varies, with numbers such as 16% [47] and 38% [48]. The presence of comorbid ADHD and BPD is associated with more severe symptoms of BPD, worse outcomes, and poor response to treatment [32, 47, 49].

The results presented in Table 5 allows us to understand how the profiles of ADHD patients differ based on SUD status in terms of psychosocial functioning. We found that the SUD group had significantly more deficits in measures of violence and educational attainment, more interactions with the law (charges), and more history of physical abuse and poor relationships with their parents. This emphasizes that the reasoning for high substance abuse and psychiatric comorbidity in the ADHD population are multifactorial. These variables may include the overlap of genetic and epigenetic vulnerabilities [30], and environmental influences such as trauma and poor social relationships. Exposure to such environmental influences may work synergistically with the neurodevelopmental influences to produce a more severe ADHD phenotype [6]. Psychiatric comorbidities and environmental adversities are high in the ADHD population, which emphasizes the importance of a personalized and tailored treatment approach that fits a patient’s biopsychosocial narrative. An individual with ADHD whom has many other comorbidities would benefit from multimodal approach that may include biological (pharmacologic treatment for ADHD and comorbid psychiatric disorders) and psychosocial (psychoeducation, psychotherapy, family therapy, motivational interviewing, peer support groups) treatments, and any specialized treatments as needed (crisis management, withdrawal management, relapse prevention). To maintain such approaches, more emphasis and health care resource allocation needs to be put on supporting such approaches to improve accessibility.

Our study has several limitations; this study is a retrospective analysis with no blinding of participants or investigators. Identified patients are allocated into independent variable groups depending on their reported substance use on questionnaires. Therefore, whether a participant fits into the “ADHD with SUD” group or “ADHD without SUD” group is dependent on the integrity of their self-reported substance use patterns on questionnaires. Due to the study’s retrospective nature, not all confounding variables were controlled for, which may include the chronicity of substance use (i.e. whether they used cannabis regularly during adolescence), age of ADHD diagnosis, and other comorbidities and social circumstances not asked about in the questionnaires.

## Conclusion

Our study served as a pilot study and is exploratory in nature to gain more background information on SUD in the ADHD population, and how patients may differ in many psychosocial variables based on SUD status. Our results suggest that more research is needed to determine the effect that certain substances may have on neuromaturation and cognition, and how substances can be a barrier to improved ADHD outcomes. Considering how the use of cannabis recently became legalized in Canada, this study has a special importance, as it further draws attention to the complexity of heavy cannabis use, and accentuates the need to further understand how cannabis use disorder interacts with cognitive functioning.

## Data Availability

Data pertaining to this study has been deidentified, and is stored on a password-protected laptop. The datasets generated and analysed during the current study are not publicly available as the public release of deidentified data was not approved by the NSHA Research Ethics Board – as that may jeopardize the privacy and confidentiality of study participants. However any particular materials are datasets that are not included in the publication, can be made available by contacting the corresponding author on reasonable request.

## Abbreviations

ADHD: Attention-Deficit/Hyperactivity Disorder
BPD: Borderline Personality Disorder
CU: Heavy Cannabis use
IVA/CPT: Integrated Auditory and Visual Continuous Performance test
SAAQ: Sustained Auditory Attention Quotient
SVAQ: Sustained Visual Attention Quotient
SUD: Substance use Disorder
